# The emergence of spontaneous activity in neuronal cultures, coherence from noise

**DOI:** 10.1186/1471-2202-14-S1-P54

**Published:** 2013-07-08

**Authors:** Javier G Orlandi, Enric Alvarez-Lacalle, Sara Teller, Jordi Soriano, Jaume Casademunt

**Affiliations:** 1 Departament d'Estructura i Constituents de la Matèria, Universitat de Barcelona, 08028 Barcelona, Spain; 2 Departament de Física Aplicada EETAC, Universitat Politècnica de Catalunya UPC, 08860 Castelldefels, Spain

## 

When neurons are grown *in vitro *and left unperturbed, they create their own network of connections and eventually reach a state where they fire simultaneously in a strikingly regular collective pattern of nearly periodic bursts [1]. Even though the spontaneous activity of neuronal networks is widely recognized as a fundamental problem in neuroscience, the mechanisms governing these bursts of spontaneous activity are not well understood. In neuronal cultures many conflicting views coexist, from burst initiation zones in 1D systems [2], to leading neurons [3] and network interactions [4] amongst others. Here we give new insights on the emergence of spontaneous activity in two-dimensional neuronal cultures and tackle this problem with two complementary approaches.

Using high-speed calcium imaging techniques in cultures derived from cortical neurons of embryonic rat brains, we show that the collective bursts of activity are in fact propagating waves of activity that are locally nucleated. The nucleation develops in specific sites of the cultures that are independent of each other and have a well defined size.

With *in silico *modelling, we elucidate the origin of the bursting activity in terms of a new phenomenon that we call noise focusing, a fast nucleation mechanism that relies both on detailed network topology and on the neurons' integrate-and-fire dynamics. The phenomenon consists of a selective, highly directional noise amplification mechanism that drives activity avalanches towards a few focal points that act as noise attractors. The resulting scenario challenges common understanding of neuronal cultures while providing a simple physical explanation of all the existing data with no appeal to any biological guidance of the network architecture.
